# From structure to sequence: Antibody discovery using cryoEM

**DOI:** 10.1126/sciadv.abk2039

**Published:** 2022-01-19

**Authors:** Aleksandar Antanasijevic, Charles A. Bowman, Robert N. Kirchdoerfer, Christopher A. Cottrell, Gabriel Ozorowski, Amit A. Upadhyay, Kimberly M. Cirelli, Diane G. Carnathan, Chiamaka A. Enemuo, Leigh M. Sewall, Bartek Nogal, Fangzhu Zhao, Bettina Groschel, William R. Schief, Devin Sok, Guido Silvestri, Shane Crotty, Steven E. Bosinger, Andrew B. Ward

**Affiliations:** 1 Department of Integrative Structural and Computational Biology, The Scripps Research Institute, La Jolla, CA 92037, USA.; 2 Consortium for HIV/AIDS Vaccine Development (CHAVD), The Scripps Research Institute, La Jolla, CA 92037, USA.; 3 Department of Biochemistry, College of Agricultural and Life Sciences, University of Wisconsin, Madison, WI 53706, USA.; 4 Department of Immunology and Microbiology, The Scripps Research Institute, La Jolla, CA 92037, USA.; 5 Department of Pathology and Laboratory Medicine, Emory School of Medicine, Emory University, Atlanta, GA 30329, USA.; 6 Yerkes Division of Microbiology and Immunology, Yerkes National Primate Research Center, and Yerkes Nonhuman Primate Genomics Core, Emory University, Atlanta, GA 30329, USA.; 7 Vaccine Discovery Division, La Jolla Institute for Immunology, La Jolla, CA 92037, USA.; 8 International AIDS Vaccine Initiative–Neutralizing Antibody Center (IAVI-NAC), The Scripps Research Institute, La Jolla, CA 92037, USA.; 9 The Ragon Institute of Massachusetts General Hospital, Massachusetts Institute of Technology and Harvard University, Cambridge, MA 02139, USA.

## Abstract

One of the rate-limiting steps in analyzing immune responses to vaccines or infections is the isolation and characterization of monoclonal antibodies. Here, we present a hybrid structural and bioinformatic approach to directly assign the heavy and light chains, identify complementarity-determining regions, and discover sequences from cryoEM density maps of serum-derived polyclonal antibodies bound to an antigen. When combined with next-generation sequencing of immune repertoires, we were able to specifically identify clonal family members, synthesize the monoclonal antibodies, and confirm that they interact with the antigen in a manner equivalent to the corresponding polyclonal antibodies. This structure-based approach for identification of monoclonal antibodies from polyclonal sera opens new avenues for analysis of immune responses and iterative vaccine design.

## Teaser

CryoEM and next-generation sequencing were used to identify monoclonal antibodies elicited by HIV Env vaccine candidates.

## INTRODUCTION

Comprehensive analyses of immune responses to infection or vaccination are laborious and expensive (*1*, *2*). Classical serology approaches, based on enzyme-linked immunosorbent assay (ELISA) and viral neutralization assays, offer a wealth of information but require a relatively large set of biological and viral reagents (*3*–*5*). For rational and structure-based vaccine design efforts, it is also necessary to isolate specific monoclonal antibodies (mAbs) elicited by a vaccine/pathogen (*6*–*11*), yet the isolation of antigen-specific B cells requires fluorescently labeled probes and access to advanced cell sorting equipment (*12*–*14*). Individual mAbs are subsequently produced and subjected to further binding and structural and functional evaluation to assess epitope specificity, affinity, and activity (i.e., neutralization capacity). High-resolution structural characterization of selected antibodies is most commonly performed at the end of this process and requires the acquisition of separate datasets for each unique sample (*1*, *15*, *16*).

Recently, we developed an approach that uses cryo–electron microscopy (cryoEM) for characterization of polyclonal antibody (pAb) responses elicited by vaccination or infection (cryoEMPEM) on the level of immune sera (*17*). From a single cryoEMPEM dataset, we can readily reconstruct maps of immune complexes at near-atomic resolution (~3- to 4-Å range), bypassing the mAb isolation steps and streamlining the structural analysis. However, the polyclonal nature of the bound antibodies and the inherent lack of sequence information restrict true atomic resolution for the reconstructed maps and thereby limit the interpretation of specific epitope-paratope contacts.

Here, we developed a method to determine mAb sequences directly from cryoEMPEM maps. This hybrid approach, consisting of electron microscopy (EM) and next-generation sequencing (NGS), enabled sequence assignment of variable regions of polyclonal Fabs (Fv) including the complementarity-determining regions (CDRs).

## RESULTS

**Fig. 1.**
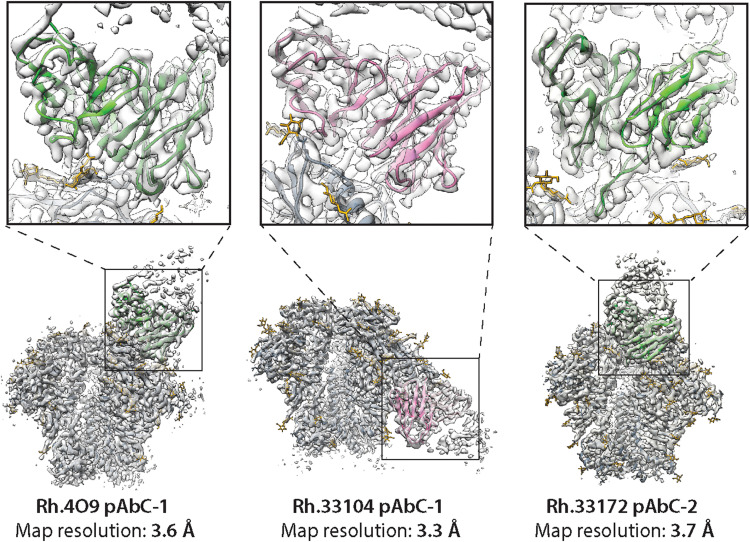
CryoEMPEM maps featuring BG505 SOSIP antigen and rhesus macaque pAbs. High-resolution maps (transparent gray surface) and corresponding models of immune complexes with pAbs (from left to right: Rh.4O9 pAbC-1, Rh.33104 pAbC-1, and Rh.33172 pAbC-2). Full maps and models are shown on the bottom, and the close-up views of the epitope-paratope interfaces are shown on top. Ribbon representation is used for models (BG505 SOSIP, dark gray; Rh.4O9 polyclonal Fab, green; Rh.33104 polyclonal Fab, pink; and Rh.33172 polyclonal Fab, green). Only the variable domains of polyclonal Fabs were modeled. N-linked glycans are shown as sticks and colored yellow.

We used structural data from the recently published rhesus macaque immunization experiments with soluble HIV Env trimers (*17*, *18*). The three primary cryoEMPEM maps/models used in this study featured BG505 SOSIP bound to structurally distinct pAbs (Fig. 1). The cryoEM maps were of excellent quality (3.3- to 3.7-Å resolution) with high local resolution for the part of the EM map corresponding to the Fab (Fig. 1 and fig. S1).

**Fig. 2.**
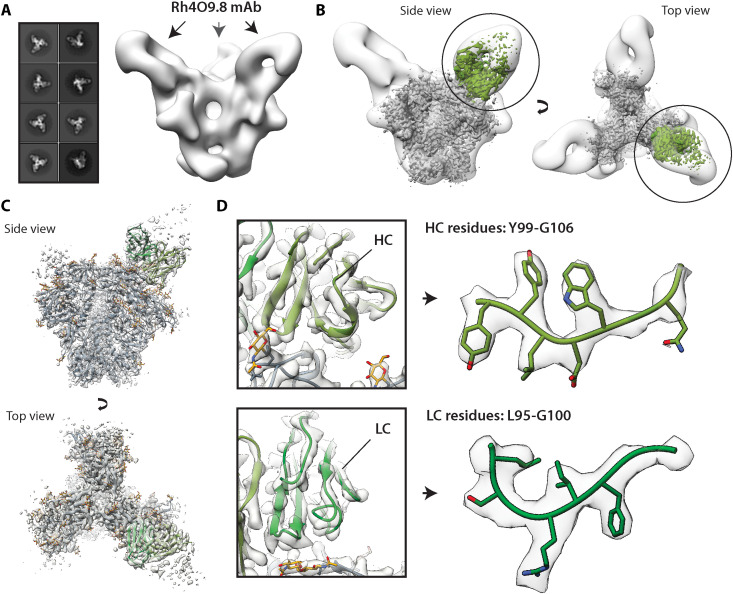
Comparison of mAb and pAb structures of an antibody that targets the V1-loop of BG505 SOSIP. (**A**) nsEM characterization of the Rh4O9.8 Fab in complex with BG505 SOSIP trimer (left: 2D class averages, right: reconstructed 3D map). The arrows show the location of the antibody. (**B**) Overlay of the nsEM map featuring Rh4O9.8 mAb (transparent white surface) and the Rh.4O9 pAbC-1 cryoEMPEM map (BG505 SOSIP, gray; pAb, green). (**C**) Ribbon representation of the atomic model of BG505 SOSIP (peptide, dark gray; glycans, yellow) and Rh4O9.8 mAb (green) complex built into the polyclonal cryoEMPEM map, Rh.4O9 pAbC-1 (transparent white surface). (**D**) Close-up view showing model-to-map fit for the heavy chain (HC; top) and the light chain (LC; bottom) of the Rh4O9.8 mAb, including parts of the HCDR3 and LCDR3 (heavy chain, olive green; light chain, forest green; and EM map, transparent white surface).

First, we analyzed the Rh.4O9 pAbC-1 cryoEMPEM map (Fig. 1, left), featuring a pAb bound to the V1 loop of BG505 SOSIP antigen. The originally published dataset (*18*) was reprocessed using the focused classification approach shown in fig. S2 to reduce heterogeneity and reconstruct higher-quality map of the V1 pAb. mAbs recognizing this epitope have recently been isolated from the same rhesus macaque (*19*). Two antibodies from the same clonal family, Rh4O9.7 and Rh4O9.8, neutralized the wild-type BG505 pseudovirus and were found to target the V1 loop through mutagenesis. We applied negative stain electron microscopy (nsEM) to characterize the binding of Rh4O9.8 antibody to BG505 SOSIP and confirmed the V1 specificity (Fig. 2A). Furthermore, the Rh4O9.8 mAb superimposed with the polyclonal Fab from the Rh.4O9 pAbC-1 map (Fig. 2B).

The sequence of Rh4O9.8 was used to build an atomic model into the Fab-corresponding part of the Rh.4O9 pAbC-1 map (Fig. 2C). The model exhibited excellent agreement with the cryoEMPEM map at the secondary structure level (Fig. 2D, left) and the side-chain level (Fig. 2D, right). Per-residue *Q* score plots (*20*) confirm good overall model-to-map fit for the Rh4O9.8 antibody with high degree of consistency between the framework (FW) and the CDR regions (fig. S3). Together, the structural data strongly suggest that the Rh4O9.8 mAb isolated by B cell sorting is a clonal relative of the V1-targeting pAbs identified at the serum level by cryoEM.

In the above example, knowing the sequence of the selected related mAbs enabled interpretation of cryoEM density at an atomic level. However, given the polyclonal nature of bound antibodies and final cryoEMPEM map resolutions of ~3 to 4 Å, the structural information is too ambiguous to directly determine antibody sequence from structure alone. We hypothesized that if appropriate sequence databases featuring the B cell receptor (BCR) repertoire at the time point that matches the time point of serum (i.e., pAb) collection were available, then the structural information from pAb-containing cryoEMPEM maps could be used to select the heavy and light chain sequence candidates from those databases. Thus, we developed a hybrid approach that uses the structural restraints from cryoEMPEM maps and NGS data of antigen-specific B cell repertoires for identification of mAbs. Full method workflow is illustrated in fig. S4.

**Fig. 3.**
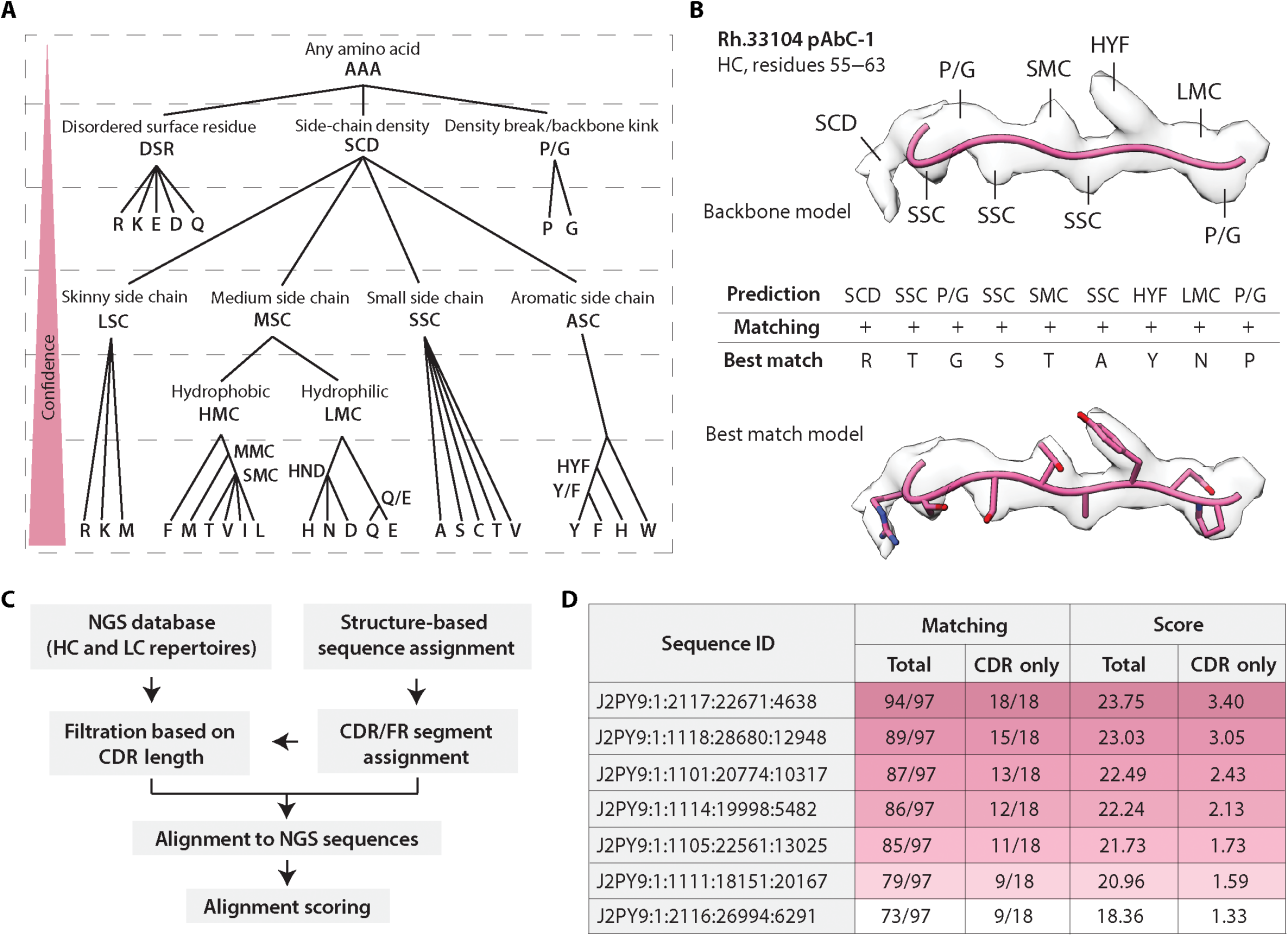
Sequence prediction from high-resolution cryoEMPEM maps. (**A**) Amino acid categories were defined on the basis of common structural features. Hierarchical system allows confidence-based assignment of amino acids and offers more flexibility in heterogeneous density maps. (**B**) Example of the amino acid assignment process using Rh.33104 pAbC-1 (heavy chain, residues 55 to 63). Category assignments are based on the structural features at each amino acid position. Models are displayed in pink, and maps are represented as transparent light gray surface. The bottom part shows the best matching sequence from the database search and the corresponding model. (**C**) Flowchart of the steps within the sequence alignment and scoring program. (**D**) Examples of matching and scoring results for a subset of NGS sequences, calculated from the search algorithm output data (Rh.33104 dataset, light chain query). The parameters are calculated for the entire aligned portion of the sequence (total) and for CDR segments without the FW (CDR-only). For clarity, the category labels are depicted by one- to three-letter abbreviations in (A) and (B). However, in the current version of the program, they are numerical (fig. S5).

First, to approximate the inherent ambiguity in interpreting 3- to 4-Å resolution cryoEM maps, we developed an assignment system that integrates the degree of certainty associated with the corresponding structural features (i.e., density volume surrounding each amino acid) (Fig. 3A and fig. S5). The assignments are performed manually with each amino acid position depicted with a hierarchical category identifier corresponding to a predefined subset of amino acid residues that best correspond to the density. An example of category-based assignment using the Rh.33104 pAbC-1 map is shown in Fig. 3B. At the end of this process, structural homology with published antibody structures is used to define the CDR and FW regions, resulting in an initial hierarchical assignment of the antibody sequence.

Next, we designed a search algorithm to take two main inputs: (i) preprocessed NGS sequence databases and (ii) the user-defined, structure-based sequence queries (Fig. 3C). NGS data are prefiltered by CDR lengths determined during the sequence assignment step by a user-set length tolerance. The program then performs a nongapped exhaustive alignment search of the query versus every sequence in the database. The queries are split by feature (FW1/2/3 and CDR1/2/3), and each feature is aligned independently. Matching is determined on the basis of the agreement of the assigned amino acid category and the corresponding amino acid from the aligned NGS sequence. For each match (depicted as “+” in the outputs) at a given position, the score is calculated on the basis of the relative ambiguity of the category (1/*X*_i_, where *X*_i_ = the number of possible amino acids, *X*, at position i). Mismatches (depicted as “−” in the outputs) are scored as 0. The output ranks matching sequences based on the CDR lengths, alignment scores, and the number and location of mismatches (if any). The calculated score and matching are the two main parameters used to evaluate the agreement of structural data with the sequences from the database (Fig. 3D).

To validate the sequence prediction algorithm, we applied it to Rh.33104 pAbC-1 and Rh.33172 pAbC-2 cryoEMPEM datasets (Fig. 1). Antibody sequence databases were generated using the germinal center B cells harvested at week 27 time point via fine-needle aspiration (*21*); the serum samples used for cryoEMPEM analysis were collected at weeks 26 and 38 from macaques Rh.33104 and Rh.33172, respectively. Extracted B cells were sorted on the basis of their binding to the BG505 SOSIP antigen (fig. S6). The sorted B cells were pooled, lysed, and subjected to the NGS, as described in Materials and Methods (see table S1 for the list of sequencing primers). Notably, the specific heavy and light chain pairing is lost during this process. The total number of sequences recovered for each query is shown in table S2.

Structure-based sequence category assignments for Rh.33104 pAbC-1 and Rh.33172 pAbC-2 are provided in auxiliary tables S1 and S2, respectively. The NGS database alignment results with scores are shown in auxiliary tables S3 to S6. During the alignment analysis, special emphasis was placed on the score within the CDR regions as the most relevant site for comparisons between different antibodies. The heavy and light chain sequence candidates with the highest scores (for the total sequence and CDRs only) and best matching to predictions were selected and evaluated. Model-to-map fits, alignment, and scoring statistics for the best matching Rh.33104 pAbC-1 and Rh.33172 pAbC-2 sequences are shown in figs. S7 and S8, respectively. The mismatches (disagreements between the assigned amino acid category and the amino acid from the best matching sequence) comprised 4 to 18% of the residues in the heavy and light chain sequences (figs. S7B and S8B); for CDRs, they occurred in 0 to 14% of cases. Examples of the most common types of mismatches are presented in fig. S9.

**Fig. 4.**
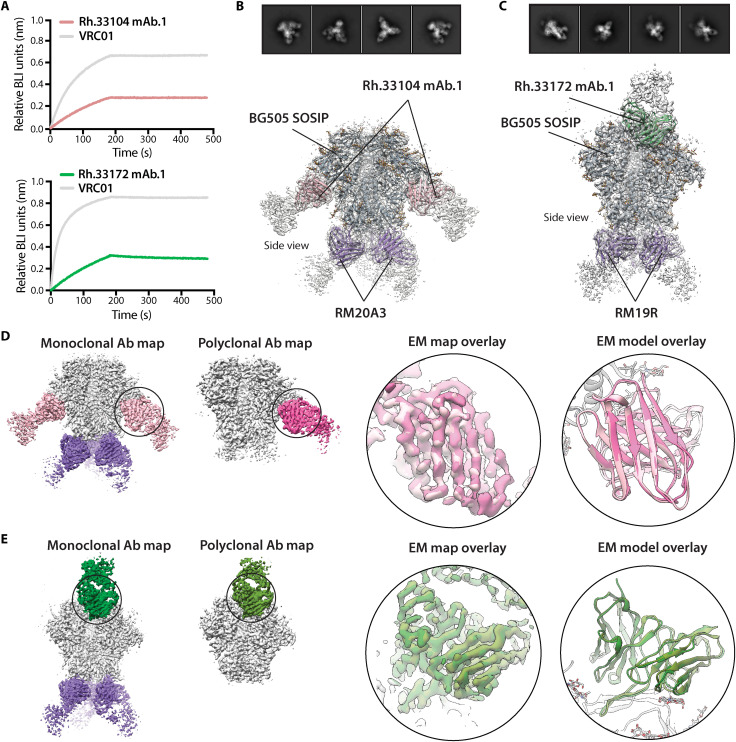
Characterization of the recovered mAbs. (**A**) Results of the BLI binding analysis performed with Rh.33104 mAb.1 (top) or Rh.33172 mAb.1 (bottom) in the form of IgG and the corresponding BG505 SOSIP trimer antigen. VRC01 IgG (gray curves) was included as a positive control and reference. (**B** and **C**) CryoEM maps and models of the BG505 SOSIP complexes with Rh.33104 mAb.1 (B) and Rh.33172 mAb.1 (C) as Fab fragments. Representative 2D class averages are displayed at the top of each panel. Maps are represented as transparent gray surface, and models are shown as ribbons (BG505 SOSIP, dark gray; RM19R and RM20A3, purple; Rh.33104 mAb.1, pink; and Rh.33172 mAb.1, green). (**D** and **E**) Overlay of the cryoEM maps/models from the EM experiments with monoclonal and polyclonal Fabs for Rh.33104 (D) and Rh.33172 (E) samples. Full maps of compared immune complexes are shown on top in each panel. Maps are segmented and colored (BG505 SOSIP, light gray; RM20A3 and RM19R, purple; Rh.33104 mAb.1, light pink; Rh.33104 pAbC-1, deep pink; Rh.33172 mAb.1, forest green; and Rh.33172 pAbC-1, olive green). Map (left) and model (right) overlays focusing on the Fab are shown in the bottom panels. On the backbone level (Cα), the root mean square deviation values between the mAb and pAb model were 0.668 and 0.721 Å for Rh.33104 and Rh.33172 datasets, respectively.

Antibodies based on the best matching heavy and light chain sequences from Rh.33104 and Rh.33172 queries were produced and assessed for binding to the BG505 SOSIP antigen using biolayer interferometry (BLI) and sandwich ELISA assays (Fig. 4 and fig. S10). Notably, both mAbs, termed Rh.33104 mAb.1 and Rh.33172 mAb.1, formed functional dimeric immunoglobulin G (IgG) and bound BG505 SOSIP as IgG (Fig. 4A and fig. S10, A and C) and as Fab fragments (fig. S10, B and D). EC_50_ values from ELISA experiments with IgGs were 1.93 and 2.64 μg/ml, and the dissociation constants (*K*_d_) from BLI were 890 and 180 nM for Rh.33104 mAb.1 and Rh.33172 mAb.1, respectively. These binding affinities are comparable to mAbs isolated from BG505-immunized rhesus macaques in published studies (*6*, *19*).

For further validation, the two antibodies (as Fabs) were independently complexed with BG505 SOSIP antigen and subjected to cryoEM analysis (table S3 and fig. S11). mAbs targeting the base of the BG505 SOSIP trimer (RM20A3 and RM19R) were used for co-complexing, as they help achieve optimal orientation distribution of particles on cryoEM grids. The final map resolutions for the Rh.33104 mAb.1 and Rh.33172 mAb.1 complexes were 3.3 and 3.5 Å, respectively. Atomic models were relaxed into the reconstructed maps (Fig. 4, B and C, and table S4). The structures revealed that both antibodies bound to the same epitopes as the pAbs computationally sorted from cryoEMPEM maps (Fig. 4, D and E). The overlay of the maps/models from experiments with monoclonal and polyclonal Fabs showed excellent agreement in both cases (right panels in Fig. 4, D and E). Together, the structural data confirmed that the mAbs selected from the NGS database were clonal members of the polyclonal families detected via cryoEMPEM.

## DISCUSSION

CryoEMPEM is a powerful tool for characterization of pAb responses elicited by vaccination or infection. In this study, we expanded the applicability of cryoEMPEM data by introducing a method for identification of functional antibody sequences from structural observations. Conceptually, our method is similar to the recently published approach for bottom-up structural proteomics (*22*), in that they both use structural information to infer protein sequences. However, our method uses a different assignment system, search algorithm, and set of scoring metrics that are specifically optimized for heterogeneous cryoEM density maps, such are the ones obtained by polyclonal epitope mapping.

This approach provides an alternative to traditional mAb discovery methods based on single B cell sorting, hybridoma, and phage display technologies (*12*–*14*). One of the rate-limiting steps with traditional methods for antibody isolation is screening mAb libraries to identify the clones with desired epitope specificity. Conversely, our approach starts with epitope information for antigen-specific pAbs. The structural data are coupled with the corresponding NGS database of antigen-specific BCR sequences to identify the underlying families of antibodies bound to the epitopes of interest. This effectively circumvents the requirement for single B cell sorting and mAb screening. Therefore, the analysis can be completed within a few weeks of sample collection, instead of a few months, allowing for a more immediate impact on (but not limited to) vaccine design, including real-time decision-making during immunizations, immunogen redesign for on- and off-target responses, and creation of probes for sorting specific B cell responses.

The structure-guided approach eliminates the need for high-resolution characterization of identified mAbs, as the data are already acquired on the polyclonal level, although subsequent biophysical and low-resolution structural evaluation should be performed for all recovered mAbs. We have demonstrated that our approach can identify the correct polyclonal families for the heavy and the light chains, but each family can have 100 to 1000 unique sequences. Consequently, there is a risk that selected heavy-light combination(s) may not be fully compatible and/or may bind in a manner slightly different than the starting pAb. These problems can be identified by in silico modeling candidate antibodies and assessing them. Alternatively, paired heavy-light chain sequencing of BCR repertoires would allow to select only functional heavy-light chain combinations and eliminate the risk of incompatibility.

Our approach requires high-quality structural data (~4 Å or better map resolutions). In addition, low-abundance and/or highly diverse classes of antibodies may result in structural information too ambiguous to correctly assign the heavy and light chain sequences. However, novel methods for BCR repertoire determination (e.g., paired heavy-light chain sequencing) and sequence database analysis (e.g., clustering of clonally related sequences) reduce the search space and the relative amount of structural information necessary to identify different pAb families. In simpler cases, it may only be necessary to determine the lengths of CDR/FW regions within the heavy and light chain to determine the antibody sequence; this can be readily achieved in maps of intermediate resolution (up to ~4.5 Å). Furthermore, cryoEM is rapidly improving in terms of throughput and resolution, which should only increase the applicability of our approach.

In this proof-of-concept study, we used lymph node (LN) B cells with specificity for the BG505 SOSIP antigen. Our method is anticipated to work with B cells from other sources (e.g., peripheral blood, spleen, bone marrow, plasma cells) and without the presorting for antigen binding. By directly imaging the serum antibodies using cryoEM, we have a proxy for abundance, affinity, and clonality. Hence, our approach will open up new doors for both the discovery of mAbs and analyzing antibody responses to infection and vaccination. The ongoing COVID-19 pandemic has highlighted the need for such robust and rapid technologies.

## MATERIALS AND METHODS

### Antigen expression and purification

Antigen expression and purification were performed as described previously (*17*). Briefly, BG505-SOSIP.v5.2(7S) N241/N289 (subcloned into a pPPI4 vector) and BG505 SOSIP MD39 (subcloned into a pHLsec vector) construct genes were expressed in 293F cells (Thermo Fisher Scientific). The proteins were purified from cell supernatants using PGT145 or 2G12 immunoaffinity chromatography. MgC1_2_ buffer (3 M) was used for protein elution from the immunoaffinity matrix. BG505 SOSIP samples were concentrated, buffer exchanged to tris-buffered saline (TBS) (Alfa Aesar), and subjected to size exclusion chromatography (SEC). HiLoad 16/600 Superdex 200 pg (GE Healthcare) running TBS buffer was used for SEC purification. Fractions corresponding to the BG505 SOSIP antigen were pooled, concentrated to 1 mg/ml, and frozen for storage.

### Rhesus macaque immunizations

The rhesus macaque immunization experiments have been reported previously (*17*). Immunogens were administered subcutaneously, divided between the right and left mid-thighs at weeks 0, 8, 24, and 36. Animals were immunized with BG505 SOSIP MD39 trimer (100 μg per dose) or BG505 SOSIP T33-31 nanoparticle (119 μg per dose) with Matrix-M (75 μg per dose; Novavax) or the ISCOM-like saponin adjuvant SMNP (750 U per dose; D. Irvine lab, Massachusetts Institute of Technology) adjuvants. Blood draws were performed biweekly. LN fine-needle aspirates (FNAs) were performed as previously described (*23*) at weeks 8, 11, 14, and 27. LN biopsies were performed between weeks 40 and 42. Animal work was performed at the Yerkes National Primate Research Center, Atlanta, GA, USA. All procedures were approved by Emory University Institutional Animal Care and Use Committee protocol 201700723. Animal care facilities are accredited by the U.S. Department of Agriculture and the Association for Assessment and Accreditation of Laboratory Animal Care International.

For structural cryoEMPEM analyses of pAb responses in animals Rh.33104 and Rh.33172, we used the serum samples from weeks 26 and 38, respectively (*17*). The reconstructed EM maps and models correspond to these time points. B cell repertoire databases were generated using the FNA samples from week 27.

### B cell sorting

Biotinylated BG505 SOSIP MD39 trimer and BG505 SOSIP.v5.2 N241/N289 trimers were generated as previously described (*17*, *23*). Biotinylated proteins were individually premixed with fluorochrome-conjugated streptavidin (Brilliant Violet 650 or Brilliant Violet 421, BioLegend) at room temperature for 20 min. BG505 SOSIP MD39-ferritin and BG505 SOSIP T33-31 nanoparticles were generated and directly conjugated to Alexa Fluor 647 (Thermo Fisher Scientific). Cells were incubated with indicated probes for 30 min at 4°C, and then surface antibodies were added and incubated for 30 min at 4°C. Cells were washed and then sorted on a BD FACSAria II. The sorting procedure is shown in fig. S6.

#### Rh.33104 (BG505 SOSIP MD39 immunized)

LN cells collected at week 27 were stained with the following probes and antibodies: BG505 SOSIP MD39 trimer–Brilliant Violet 650, BG505 SOSIP MD39 trimer–Brilliant Violet 421, BG505 SOSIP MD39–ferritin nanoparticle–Alexa Fluor 647, fixable viability dye eFluor 780 (Thermo Fisher Scientific), mouse anti-human CD4 APC (allophycocyanin) eFluor 780 (SK3, Thermo Fisher Scientific), mouse anti-human CD8 APC eFluor 780 (RPA-T8, Thermo Fisher Scientific), mouse anti-human CD16 APC eFluor 780 (ebioCB16, Thermo Fisher Scientific), mouse anti-human CD20 Alexa Fluor 488 (2H7, BioLegend), mouse anti-human IgG PE (phycoerythrin)–Cy7 (G18-145, BD Biosciences), mouse anti-human IgM PerCP-Cy5.5 (G20-127, BD Biosciences), mouse anti-human CD38 PE [OKT10, Nonhuman Primate (NHP) Reagent Resource], and mouse anti-human CD71 PE-CF594 (L01.1, BD Biosciences).

#### Rh.33172 (BG505 SOSIP T33-31 nanoparticle immunized)

LN cells collected at week 27 were stained with the following probes and antibodies: BG505 SOSIP MD39 trimer–Brilliant Violet 650, BG505 SOSIP.v5.2 N241/N289 trimer–Brilliant Violet 421, BG505 SOSIP T33-31 nanoparticle–Alexa Fluor 647, fixable viability dye eFluor 780 (Thermo Fisher Scientific), mouse anti-human CD4 APC eFluor 780 (SK3, Thermo Fisher Scientific), mouse anti-human CD8 APC eFluor 780 (RPA-T8, Thermo Fisher Scientific), mouse anti-human CD16 APC eFluor 780 (ebioCB16, Thermo Fisher Scientific), mouse anti-human CD20 Alexa Fluor 488 (2H7, BioLegend), mouse anti-human IgG PE-Cy7 (G18-145, BD Biosciences), mouse anti-human IgM PerCP-Cy5.5 (G20-127, BD Biosciences), mouse anti-human CD38 PE (OKT10, NHP Reagent Resource), and mouse anti-human CD71 PE-CF594 (L01.1, BD Biosciences).

### NHP immunoglobulin repertoire library preparation and sequencing

Immunoglobulin repertoire sequencing libraries were prepared using a protocol provided by D. Douek, National Institute of Allergy and Infectious Diseases/Vaccine Research Center (*24*) and similar to that described previously (*23*). Primer sequences are listed in table S1. With the exception of the Oligo dT and SMARTer II A Oligo template switch primer, all oligos were obtained from Integrated DNA Technologies. QIAGEN RNeasy kits (Valencia, CA) were used to isolate RNA from isolated antigen-specific B cells obtained from LN FNAs at the postvaccine time points described above. cDNA was generated using Clontech SMARTer kits: 8 μl of RNA was mixed with 1 μl of 5′ coding sequence oligo(dT) (12 μM) and incubated at 72°C for 3 min and 4°C for 1 minute. Following the addition of 8.5 μl of reverse transcription (RT) master mix composed of 5× RT Buffer [250 mM tris-HCl (pH 8.3), 375 mM KCl, and 30 mM MgCl_2_], dithiothreitol (DTT; 20 mM), dNTP (deoxyribonucleoside triphosphate) mix (10 mM), RNAseOUT (40 U/μl), SMARTer II A oligo (12 μM), and Superscript II RT (200 U/μl), samples were incubated at 42°C for 90 min and 70°C for 10 min. AMPure XP beads (catalog no. A63882) were used for purifying first-strand cDNA. To amplify IgG, IgK, or IgL variable regions from cDNA, we used the KAPA Real-Time Library Amplification Kit (catalog no. KK2702). The master mix was made up of 2× KAPA polymerase chain reaction (PCR) Master Mix, 5PIIA (12 μM/μl), and 5 μl of IgG, IgK, or IgL constant region primers (2 μM) (*25*). cDNA (19.3 μl) and 30.7 μl of master mix were mixed followed by centrifugation at 2000 relative centrifugal force for 1 min. This was followed by monitoring the real-time PCR. The PCR was stopped in the exponential phase (23 cycles for IgG and ~18 cycles for IgK/IgL), and AMPure XP beads were used for purification of the amplified products. Barcodes and Illumina adapters were added using two subsequent rounds of PCR. In the first step (addition of barcodes), grafting primers (forward P5_Seq BC_XX 5PIIA, 1 μl of 10 μM stock; reverse P7_i7_XX_Ig, 1 μl of 10 μM stock) containing randomized stretch of four to eight random nucleotides and unique barcodes were mixed with 46 μl of master mix (2× KAPA PCR Master Mix 2×, SYBR Green 1:10 K, and nuclease-free water) and 2 μl of 1:10 diluted IgG/IgK/IgL amplicon; mixtures were amplified using real-time PCR (seven cycles for IgG and six cycles for IgK/IgL) and purification of the library using AMPure XP Beads. The final step involved the grafting of Illumina adapters, for which 34 μl of master mix (2× KAPA PCR Master Mix, 10 μM P5_Graft P5_seq, and nuclease-free water) was mixed with 1 μl of 10 μM P7_I7_XX Ig constant region primer and 15 μl of purified product from the previous, and amplification by real-time PCR (six cycles) and final purification of the library with AMPure XP beads. Agilent bioanalyzer was used to assess the quality of the libraries. The libraries were then pooled, and the sequencing was carried out on an Illumina MiSeq (309 paired end).

### NGS data processing and sequence analysis

Read pairs were assembled and filtered for length and quality using the REpertoire Sequencing TOolkit (*26*). Sequence regions corresponding to the primers were masked, and duplicate sequences were collapsed (*26*). Germline antibody gene assignment was conducted with IgBLAST using a comprehensive Indian origin RM germline BCR database (*6*, *27*). IgBLAST results were filtered for productive sequences and sorted based on feature lengths. For heavy chain queries, a single NGS database is used for each antibody. For light chains, the search is performed independently for the Ig-κ and Ig-λ databases.

### cryoEMPEM maps and models used in the analysis

#### Rh.4O9 pAbC-1

The Rh.4O9 pAbC-1 map was generated by applying the focused classification approach (*17*) to the Rh.4O9 cryoEMPEM dataset that was published previously (*18*). These data were reprocessed to resolve a map of higher quality (i.e., higher local resolution for the Fab). The data processing workflow is illustrated in fig. S2. The resulting EM map for Rh.4O9 pAbC-1 (Fig. 1 and fig. S1) has been uploaded to the EM Data Bank (EMDB), entry identification (ID): EMD-23779. The structural model of the complex, comprising BG505 SOSIP.v5.2 (*28*) and Rh4O9.8 mAb (*19*), was built into the EM map. ABodyBuilder (*29*) was applied to create an initial model of the Fab. Previously published structure of BG505 SOSIP [Protein Data Bank (PDB) ID: 5CEZ (*30*)] was used as a starting model for the antigen. The final model of the complex was generated by applying iterative rounds of manual refinement in Coot (*31*) and automated refinement in Rosetta (*32*). For validation, we applied EMRinger (*33*) and MolProbity (*34*) software packages (table S4). Per-residue characterization of the model to map fit (shown in fig. S3) was performed in UCSF Chimera (*35*) using the *Q* score plugin (*20*). The refined model of the complex was submitted to the PDB, entry ID: 7MDT. HxB2 numbering was used for the BG505 SOSIP antigen, and Kabat numbering was applied for Rh4O9.8 antibody.

#### Rh.33104 pAbC-1 and Rh.33172 pAbC-2

The assignment of pAb sequences was performed using previously published cryoEMPEM data (*17*). Specifically, for Rh.33104 pAbC-1 and Rh.33172 pAbC-2, we used the maps uploaded under EMDB IDs: EMD-23227 and EMD-23232, respectively. The corresponding structures were previously uploaded under PDB IDs: 7L8A and 7L8F, respectively. In the structures, the polypeptide backbone of each antibody was represented as a polyalanine pseudomodel. The number of amino acid residues was adjusted to achieve the most optimal model-to-map fit on the backbone level.

### Antibody sequence assignment

Sequence assignment of the heavy and light chains was performed manually in Coot using the models and maps from the Rh.33104 pAbC-1 and Rh.33172 pAbC-2 datasets. We developed a system for amino acid assignment based on the corresponding structural features, which also takes into consideration the degree of certainty associated with each assignment. Density volume surrounding each amino acid is attributed a hierarchical category identifier, representing a predefined subset of amino acid residues that best correspond to the density. The category assignment tree is shown in fig. S5. In the final output, the heavy and light chain sequences are represented as strings of numerical category identifiers (for examples, see auxiliary tables S1 and S2). In addition to shape properties, our system allows to further categorize certain amino acid groups (e.g., medium side-chain group) based on the local environment (i.e., hydrophobic/hydrophilic), which helps narrow down the list of possible amino acids. Structural homology with published rhesus macaque antibody structures [PDB IDs: 4KTE, 4KTD, 4RFE, 4Q2Z (*36*–*38*)] is applied at the end of the assignment process to define the CDR and FW regions within the antibody.

### Sequence alignment and scoring

Sequence match searching was performed using Python 3.6.3 (www.python.org) in a Jupyter Notebook (www.jupyter.com) environment. To prepare for alignment, the hierarchical ambiguity codes from Fig. 3A were first translated into numerical codes and organized into a recursive binary tree. This allows the algorithm to call upon any specific branch and fetch all downstream amino acid possibilities in an efficient manner. The search can be performed on two main data formats: protein FASTA files and .tsv formatted output from IGBLASTn results. The search can also compare the ambiguity codes directly to the allele database present in the Indian origin rhesus macaque Germ Line Database (GLD) currently hosted by the Ward Lab (ward.scripps.edu/gld) (*6*). Search results against FASTA files and the GLD are basic and do not return information outside of alignment of the main section of sequence. Searches run on .tsv files from IGBLASTn (referenced below as “data frame”) allow the user to pass queries in a split method based on IMGT (International ImMunoGeneTics information system) designations for relevant FW and CDR loop regions (*27*, *39*). For the main searches featured here, query strings of ambiguity codes were manually segmented into the following regions: FW1, CDR1, FW2, CDR2, FW3, and CDR3. Before searching, sequences flagged as unproductive by IGBLASTn are removed, and the data frame to be searched is subjected to filtering based on user-defined criteria. The user can filter out entries from the data frame based on any column that exists, typically restricting based on a range of desired lengths of FW and CDR regions. Once the data frame is prepared and a search is initiated, the algorithm exhaustively compares each query segment to the relevant column from the data frame in pairwise fashion. Alignment is allowed to shift by a factor defined by the user (default 2 AA), and results are returned on the basis of a maximum alignment score for each query to each subject sequence. Scores are calculated as the inverse of the number of amino acids represented by each ambiguity code at each position, but only if there is a match. For example, if an ambiguity code represents a branch that has five possible amino acids and there is a match, then the position is assessed a score of 1/5 or 0.2. Scores range from 1/20 (0.05) to 1/1 (1.00). Scores are tallied for each query section of each row of the data frame and returned to the user in csv format for easy manipulation and selection of high scoring alignments. The sequence alignment program is available on Code Ocean (DOI: 10.24433/CO.9600319.v1). It will also be released on GitHub (https://github.com/) following the publication of this manuscript.

### Analysis of sequence alignment results

For search result analysis, we calculated the alignment scores for the entire sequence (total score) and the CDRs (CDR-only score). The heavy and light chain sequences featuring a combination of the highest total and CDR-only scores from each search were selected for subcloning and expression. For analysis of the light chain alignment results, we have also calculated the mean total alignment scores for all NGS sequences in the corresponding Ig-κ and Ig-λ queries. The comparison of maximum and mean total alignment scores from the two queries was applied to determine whether the antibody light chain was Ig-κ or Ig-λ. For sequences and alignment scores, see auxiliary tables S3 to S6.

### Antibody expression and purification

Top-scoring heavy and light chain sequences from the corresponding NGS database searches were subcloned into the AbVec-hIgG1 and AbVec-hIgKappa expression vectors, respectively (*40*, *41*). Sanger sequencing was applied to verify the final DNA vectors. Heavy chain (500 μg) and 250 μg of the light chain DNA expression vectors were applied for cotransfection of 1 liter of human embryonic kidney 293F cells to produce Rh.33104 mAb.1 and Rh.33172 mAb.1 (as full IgG and Fab fragments). Polyethylenimine (PEI MAX, Polysciences Inc.) was used as a transfection reagent at threefold mass excess with respect to the total DNA amount. Antibodies were purified from cell supernatants using the MAbSelect Xtra (GE Healthcare Life Sciences) and CaptureSelect IgG-CH1 (Thermo Fisher Scientific) columns for IgG and Fab purification, respectively. Antibody samples were concentrated, buffer exchanged to TBS buffer (Alfa Aesar), and then subjected to SEC purification (HiLoad 16/600 Superdex S200 pg column; GE Healthcare Life Sciences).

### nsEM of BG505 SOSIP in complex with Rh4O9.8 mAb

Rh4O9.8 mAb (*19*) was provided by the D. Sok lab (International AIDS Vaccine Initiative–Neutralizing Antibody Center, La Jolla, CA, USA). Fifteen micrograms of BG505 SOSIP MD39 was incubated with 15 μg of the Rh4O9.8 antibody (as Fab fragment) for 1 hour at room temperature. The complex was then subjected to SEC purification (Superose 6 Increase column, 10/300 GL, GE healthcare) with TBS [10 mM tris-HCl and 150 mM NaCl (pH 7.4)] as the running buffer. Fractions corresponding to the immune complex were pooled and concentrated using an Amicon filter unit with 10-kDa cutoff (EMD Millipore). For nsEM, the SOSIP-Fab complex was diluted to 20 μg/ml, and 3 μl was applied onto a carbon-coated copper grid (400 mesh, Electron Microscopy Sciences). The grid was pretreated by glow discharge for 30 s. The complex solution was blotted off after 10 s, and the grid was subsequently stained with 2% (w/v) uranyl formate for 60 s. Imaging was performed on a Tecnai F20 electron microscope operating at 200 keV (1.77 Å per pixel; ×62,000 magnification) as described previously (*42*). The defocus was set to −1.50 μm, and the electron dose was adjusted to 25 *e*^−^/Å^2^ using Leginon software (*43*). All two-dimensional (2D) and 3D classification and 3D refinement steps were conducted in Relion 3.0 (*44*). EM density maps were visualized in UCSF Chimera (*35*). Reconstructed EM map of BG505 SOSIP in complex with three Rh4O9.8 Fabs was submitted to EMDB (ID: EMD-23778).

### Sandwich ELISA assays

Sandwich ELISA experiments were performed with Rh.33104 mAb.1 and Rh.33172 mAb.1 (as IgG). All experiments were performed in triplicates. BioStack Microplate Stacker system (BioTek) was used for buffer addition and wash steps. Antibody (12 N) with specificity toward the base of BG505 SOSIP was diluted to 3 μg/ml and immobilized onto high-binding, 96-well microplates (Greiner Bio-One) for 2 hours at room temperature. The plates were washed three times with TBST buffer (TBS + 0.1% Tween 20) and blocked overnight with TBS + 5% bovine serum albumin (BSA) + 0.05% Tween 20 at 4°C. Plates were washed three times with TBST, followed by the addition of the antigen solution [phosphate-buffered saline (PBS) + 1% BSA + BG505 SOSIP (3 μg/ml)]. For Rh.33172 mAb.1 experiments, we used BG505 SOSIP.v5.2(7S) N241/N289 construct; for Rh.33104 mAb.1 experiments, we used BG505 SOSIP MD39. This was done to match the BG505 SOSIP construct to the original immunogen that elicited the corresponding pAbs in rhesus macaques (*17*). The plates were incubated with antigen solution for 2 hours and then washed three times with TBST. Serial threefold dilutions of Rh.33104 mAb.1 or Rh.33172 mAb.1 (starting at 100 μg/ml) were prepared in TBS and added to plates coated with corresponding antigen. The plates were incubated for 2 hours at room temperature and subsequently washed three times with TBST. AP-conjugated AffiniPure goat anti-human IgG (Jackson ImmunoResearch, cat. no. 109-055-097) was diluted 1:4000 in TBS + 1% BSA buffer and added to each well for 1 hour at room temperature. Following three wash steps with TBST, 1-Step PNPP (p-nitrophenyl phosphate) Substrate Solution (Thermo Fisher Scientific) was applied to each well for detection. Synergy H1 plate reader (BioTek) was used for acquisition of colorimetric data by recording the absorbance at 405-nm wavelength. Data were analyzed in GraphPad Prism (version 8.4.3) software, and midpoint titers (EC_50_) were determined.

### Biolayer interferometry

Octet RED96 instrument (FortéBio) was used for BLI data collection. Antibody and antigen solutions were prepared in kinetics buffer [Dulbecco’s PBS + 0.1% (w/v) BSA + 0.02% (v/v) Tween 20]. All BLI experiments were conducted at 25°C. BLI experiments with IgGs were performed as described previously (*17*, *45*). Rh.33104 mAb.1, Rh.33172 mAb.1, and VRC01 antibodies (as IgGs) were diluted to 5 μg/ml and immobilized onto human anti-hIgG Fc capture (AHC) biosensors (FortéBio). VRC01 served as a positive control. Antibody-coated sensors were then transferred to wells with corresponding BG505 SOSIP antigens (see “Sandwich ELISA assays” section above for explanation) at 1000-nM concentration. Association and dissociation steps were set to 180 and 300 s, respectively. Data were processed using Octet System Data Analysis v9.0 (FortéBio). Negative control measurements (with kinetics buffer) were used for background correction. Final plots were prepared in GraphPad Prism (version 8.4.3).

Experiments with Fab fragments were performed as described previously (*6*). Fabs were diluted to 25 μg/ml and immobilized onto anti-human Fab-CH1 (FAB2G) biosensors (FortéBio). Serial twofold dilutions of the corresponding BG505 SOSIP antigens (see “Sandwich ELISA assays” section above for explanation) were prepared for binding studies, starting at 2000 nM. The lengths of association and dissociation steps were set to 600 and 1200 s, respectively. Data processing and determination of kinetic parameters were performed in Octet System Data Analysis v9.0 software (FortéBio). Data plots were prepared in GraphPad Prism (version 8.4.3).

### CryoEM analysis of mAb complexes: Preparation of complexes

#### Rh.33104 mAb.1 complex preparation

BG505 SOSIP MD39 (250 μg) was incubated with 600 μg of Rh.33104 mAb.1 Fab and 600 μg of RM20A3 Fab (*6*, *46*) at room temperature overnight. The complex was SEC purified using a HiLoad 16/600 Superdex pg200 (GE Healthcare) column, with TBS as a running buffer. SEC fractions corresponding to the complex were pooled and concentrated to 6.0 mg/ml using an Amicon filter unit with 10-kDa cutoff (EMD Millipore).

#### Rh.33172 mAb.1 complex preparation

BG505 SOSIP.v5.2(7S) N241/N289 (250 μg) was incubated with 600 μg of Rh.33172 mAb.1 Fab and 600 μg of RM19R Fab (*6*) at room temperature overnight. All other purification steps were equivalent as with Rh.33104 mAb.1 complex.

Antibodies RM20A3 and RM19R, with specificity toward the trimer base, were used for cocomplexing because in the past, we have observed that they can improve the orientational distribution of HIV Env trimers on cryoEM grids (*46*).

### CryoEM analysis of mAb complexes: Grid preparation

UltrAuFoil R 1.2/1.3 grids (Au, 300 mesh; Quantifoil Micro Tools GmbH) were used for sample vitrification. The grids were treated with Ar/O_2_ plasma (Solarus 950 plasma cleaner, Gatan) for 10 s immediately before sample application. A total of 0.5 μl of 0.04 mM lauryl maltose neopentyl glycol stock solution was mixed with 3.5 μl of the complex, and 3 μl was immediately loaded onto the grid. Grids were prepared using Vitrobot mark IV (Thermo Fisher Scientific). Temperature inside the chamber was maintained at 10°C, while humidity was at 100%. Blotting force was set to 0, wait time to 10 s, while the blotting time was varied within a 4- to 7-s range. Following the blotting step, the grids were plunge frozen into liquid ethane and cooled by liquid nitrogen.

### CryoEM analysis of mAb complexes: Data collection and processing

Samples were imaged on an FEI Titan Krios electron microscope (Thermo Fisher Scientific) operating at 300 keV. The microscope was equipped with the K2 summit detector (Gatan) operating in counting mode. Exposure magnification was 29,000, and the pixel size was 1.03 Å (at the specimen plane). Leginon software suite (*43*) was used for automated data collection. Data collection information for the two datasets featuring different mAb complexes is presented in table S3. Micrograph movie frames were aligned and dose weighted using MotionCor2 (*47*), and Gctf (*48*) was applied for estimation of CTF parameters. Initial processing steps (particle picking and 2D classification) were performed in cryoSPARC.v2 (*49*). Ab initio refinement in cryoSPARC was applied to generate the initial reference model for each complex. Clean particle stack was subsequently transferred to Relion 3.0 (*44*) for further 2D and 3D processing steps. Data processing workflows and relevant information are presented in fig. S11.

### CryoEM analysis of mAb complexes: Model building and refinement

Postprocessed cryoEM maps from Relion 3.0 were used to generate atomic models. PDB entry 6vfl (*50*) was used as initial model for BG505 SOSIP corresponding part of the complex. The sequence was adjusted to match the exact BG505 SOSIP variant used for the preparation of imaged mAb complex [BG505 SOSIP MD39 or BG505 SOSIP.v5.2(7S) N241/N289]. Initial models for RM20A3 and RM19R antibodies were adapted from PDB 6X9R (*46*) and PDB 6VKN (*6*), respectively. ABodyBuilder (*29*) was applied to create the initial Fab models for Rh.33104 mAb.1 and Rh.33172 mAb.1. Individual components were docked into the corresponding parts of each cryoEM map in UCSF Chimera (*35*) to create the initial atomic models. Iterative rounds of manual model refinement in Coot (*31*) and automated refinement using Rosetta (*32*) were performed to produce the final models. HxB2 numbering was used for BG505 SOSIP antigens, and Kabat numbering was applied for antibodies in each complex. For model validation, we applied EMRinger (*33*) and MolProbity (*34*) packages. Model refinement statistics is reported in table S4. The refined models were submitted to the PDB (ID: 7MDU and 7MEP).

## Supplementary Materials

### This PDF file includes:

Legends for Auxiliary Tables S1 to S6 Tables S1 to S4 Figs. S1 to S11

### Other Supplementary Material for this manuscript includes the following:

Auxiliary Tables S1 to S6

## Notes

View/request a protocol for this paper from *Bio-protocol*.
