# Supplementary material for: From structure to sequence: Antibody discovery using cryoEM
Source: Sci Adv. 2022 Jan 19;8(3):eabk2039. doi: 10.1126/sciadv.abk2039 (PMC8769551; doi:10.1126/sciadv.abk2039)
Supplement: Supplementary file 1 — Legends for Auxiliary Tables S1 to S6 Tables S1 to S4 Figs. S1 to S11 [file sciadv.abk2039_sm.pdf]

Science Advances
AAAS

Supplementary Materials for

From structure to sequence: Antibody discovery using cryoEM

Aleksandar Antanasijevic, Charles A. Bowman, Robert N. Kirchdoerfer, Christopher A. Cottrell, Gabriel Ozorowski, Amit A. Upadhyay, Kimberly M. Cirelli, Diane G. Carnathan, Chiamaka A. Enemuo, Leigh M. Sewall, Bartek Nogal, Fangzhu Zhao, Bettina Groschel, William R. Schief, Devin Sok, Guido Silvestri, Shane Crotty, Steven E. Bosinger, Andrew B. Ward*

*Corresponding author. Email: andrew@scripps.edu

Published 19 January 2022, *Sci. Adv.* **8**, eabk2039 (2022)
DOI: 10.1126/sciadv.abk2039

The PDF file includes:

Legends for Auxiliary Tables S1 to S6
Tables S1 to S4
Figs. S1 to S11

Other Supplementary Material for this manuscript includes the following:

Auxiliary Tables S1 to S6

Supplementary Figures and Tables

Auxiliary Supplementary Table 1. Structure-based sequence assignment for Rh.33104 pAbC-1 (provided externally)
Auxiliary Supplementary Table 2. Structure-based sequence assignment for Rh.33172 pAbC-2 (provided externally)
Auxiliary Supplementary Table 3. Search results for Rh.33104 light chain (Ig-κ) dataset (provided externally)
Auxiliary Supplementary Table 4. Search results for Rh.33104 heavy chain dataset (provided externally)
Auxiliary Supplementary Table 5. Search results for Rh.33172 light chain (Ig-κ) dataset (provided externally)
Auxiliary Supplementary Table 6. Search results for Rh.33172 heavy chain dataset (provided externally)

Table S1. Primer sequences used for NHP IgG/IgK/IgL repertoire sequencing.

Primer	Sequence
**CDS Oligo dT**	TTTTTTTTTTTTTTTTTTTTTTTTTVN
**SMARTer II A Oligo**	AAGCAGTGGTATCAACGCAGAGTACATrGrGrG
**IgG**	CCAGGGGGAAGACCGATGGGCCCTTGGTGGA
**IgK**	GCGGGAAGATGAAGACAGATGGTGCAGCCACAG
**IgL**	GGCCTTGTTGGCTTGAAGCTCCTCAGAGGAGGG
**P5_Seq BC_XX 5PIIA**	CACGACGCTCTTCCGATCT NNNN AACCACTA AAGCAGTGGTATCAACGCAGAGT
**P7_ i7_XX IgG**	CAAGCAGAAGACGGCATACGAGAT TAGTGGTT GCCAGGGGGAAGACCGATGGGCCCTTGGTGGA
**P7_ i7_XX IgK**	CAAGCAGAAGACGGCATACGAGAT TAGTGGTT GCGGGAAGATGAAGACAGATGGTGCAGCCACAG
**P7_ i7_XX IgL**	CAAGCAGAAGACGGCATACGAGAT TAGTGGTT GGCCTTGTTGGCTTGAAGCTCCTCAGAGGAGGG
**P5_Graft P5_seq**	AATGATACGGCGACCACCGAGATCTACAC TCTTTCCCTACACGACGCTCTTCCGATCT

Table S2. Sequence count for different NGS datasets

Sequence count	Rh.33104 Ig-κ	Rh.33104 Ig-λ*	Rh.33104 IgH	Rh.33172 Ig-κ	Rh.33172 Ig-λ*	Rh.33172 IgH
**Starting NGS dataset**	178299	209168	137916	192252	235499	197099
**Filtered by CDR lengths**	70845	42185	5578	117254	111770	4428
**Selected**	1	-	1	1	-	1

* Ig-λ datasets were used in the searches but in both cases the average and maximum scores were significantly lower compared to Ig-κ. Therefore Ig-λ sequences were not considered further.

Table S3. CryoEM data collection information

	BG505 SOSIP + Rh.33104 mAb.1 + RM20A3	BG505 SOSIP + Rh.33172 mAb.1 + RM19R
**Microscope**	Titan Krios	Titan Krios
**Voltage (kV)**	300	300
**Detector**	Gatan K2 Summit	Gatan K2 Summit
**Recording mode**	Counting	Counting
**Magnification**	29,000 X	29,000 X
**Movie micrograph pixel size**	1.03	1.03
**Dose rate (e^{-}/Å^2/s)**	4.70	4.70
**No. of frames per movie micrograph**	38	38
**Frame exposure time (ms)**	250	250
**Movie micrograph exposure time (s)**	9.5	9.5
**Total dose (e^{-}/Å^2)**	44.7	44.7
**Under focus range (μm)**	0.8 – 1.6	0.7 – 1.6
**Number of movie micrographs**	1022	2050

Table S4. Model building and refinement information

	BG505 SOSIP + Rh.4O9 pAbC-1	BG505 SOSIP + Rh.33104 mAb.1 + RM20A3	BG505 SOSIP + Rh.33172 mAb.1 + RM19R
**EMDB ID**	EMD-23779	EMD-23780	EMD-23801
**Map Resolution (Å)**	3.6	3.3	3.5
**Map Symmetry**	C1	C3	C1
**PDB ID**	7MDT	7MDU	7MEP
**Residues**	1970	1051	2686
**Amino-acids**	1892	1018	2596
**Carbohydrates**	78	33	90
**RMSD Bonds**	0.022	0.023	0.023
**RMSD Angles**	1.771	1.711	1.696
**Ramachandran**			
**Outliers (%)**	0.00	0.00	0.00
**Allowed (%)**	2.49	2.10	1.49
**Favored (%)**	97.51	97.90	98.51
**Rotamer outliers**	0.12	0.00	0.00
**Clash score**	0.63	1.04	0.89
**Molprobity score**	0.81	0.83	0.77
**EMRinger score**	3.38	4.32	3.66

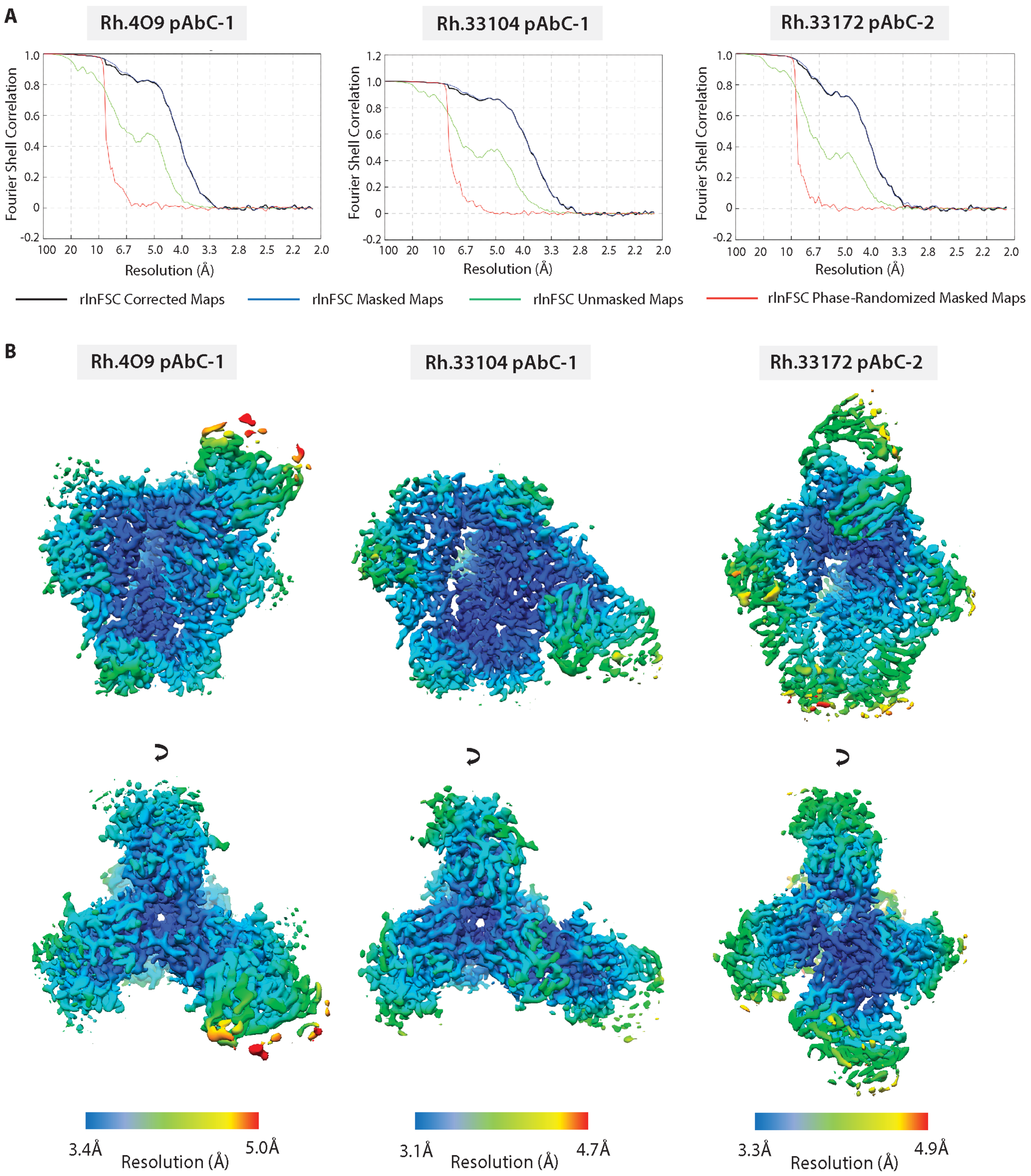

Figure S1. [A] Fourier shell correlation curves for trimer-pAbC complexes obtained using cryoEMPEM. **[B]** Local resolution plots for the trimer-pAbC complexes under investigation. The data for Rh.33172 pAbC-2 and Rh.33104 pAbC-1 were adapted from previously published work (17).

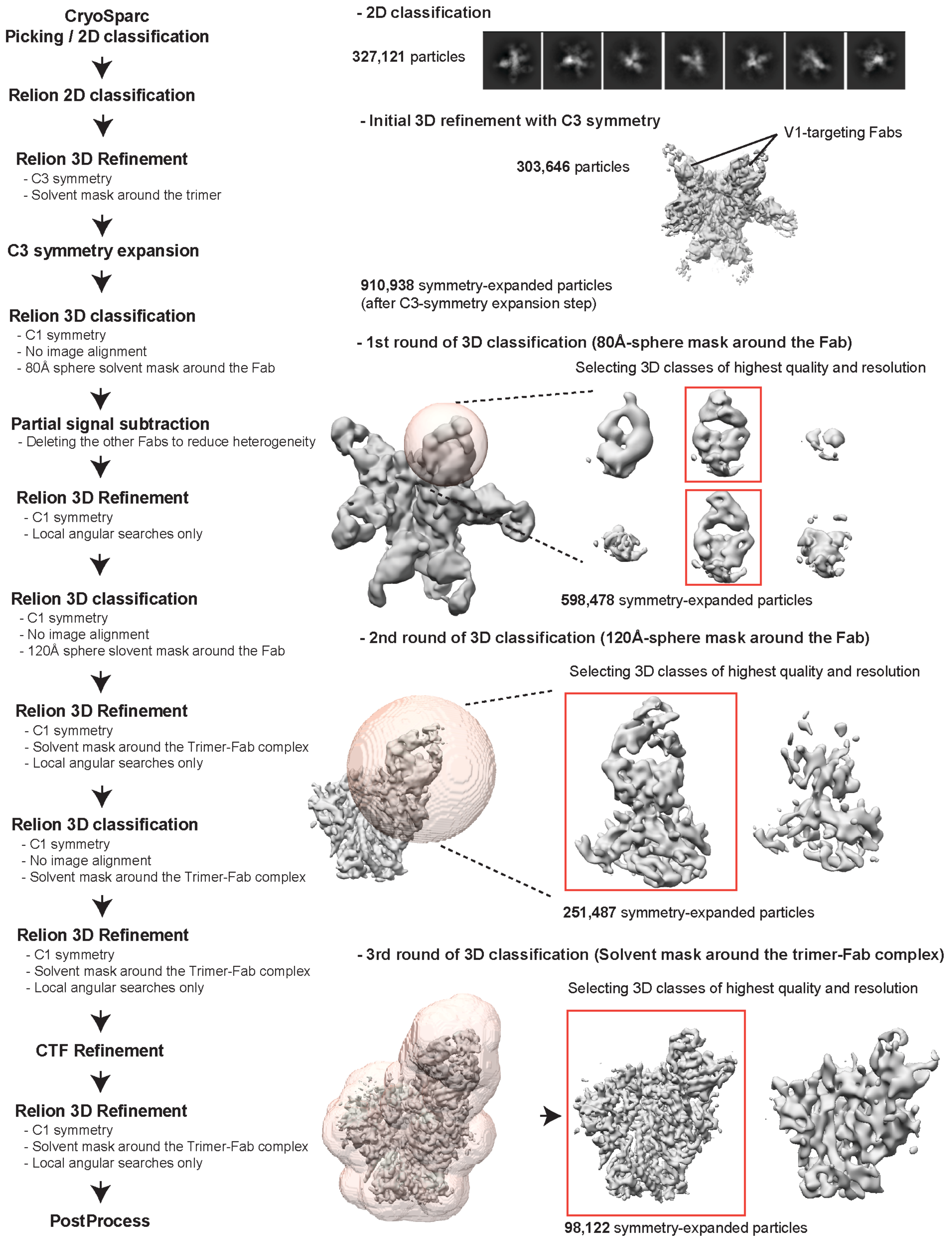

Figure S2. Schematic representation of the data processing workflow for Rh.4O9 cryoEMPEM data. Intermediate results and particle count data are shown on the right.

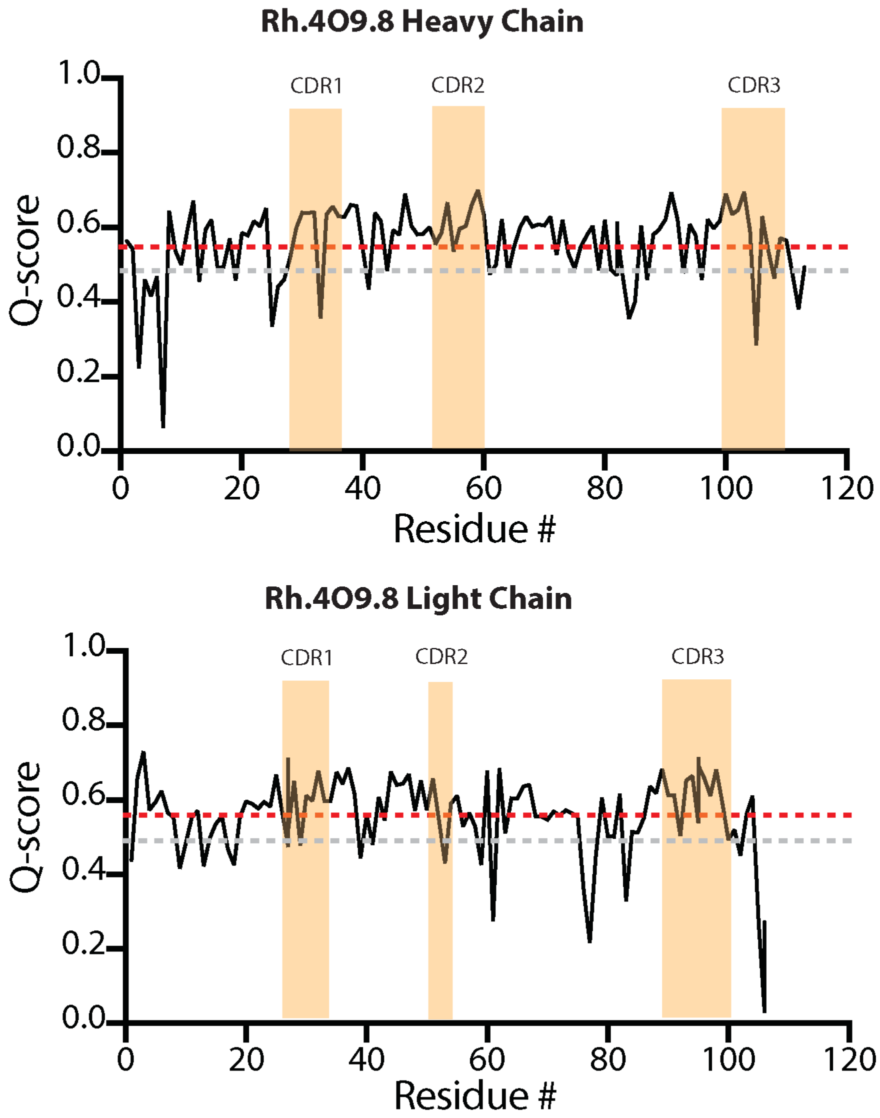

Figure S3. Analysis of the model-to-map fit for the Rh.4O9.8 antibody and the Rh.4O9 pAbC-1 cryoEMPEM map. Per-residue Q-score values for the heavy (top) and light (bottom) chains of the Rh.4O9. Average Q-score for each chain is displayed as dotted red line. The expected average Q-score for a map of 3.6 Å resolution is shown as a dotted gray line. CDRs are represented in orange.

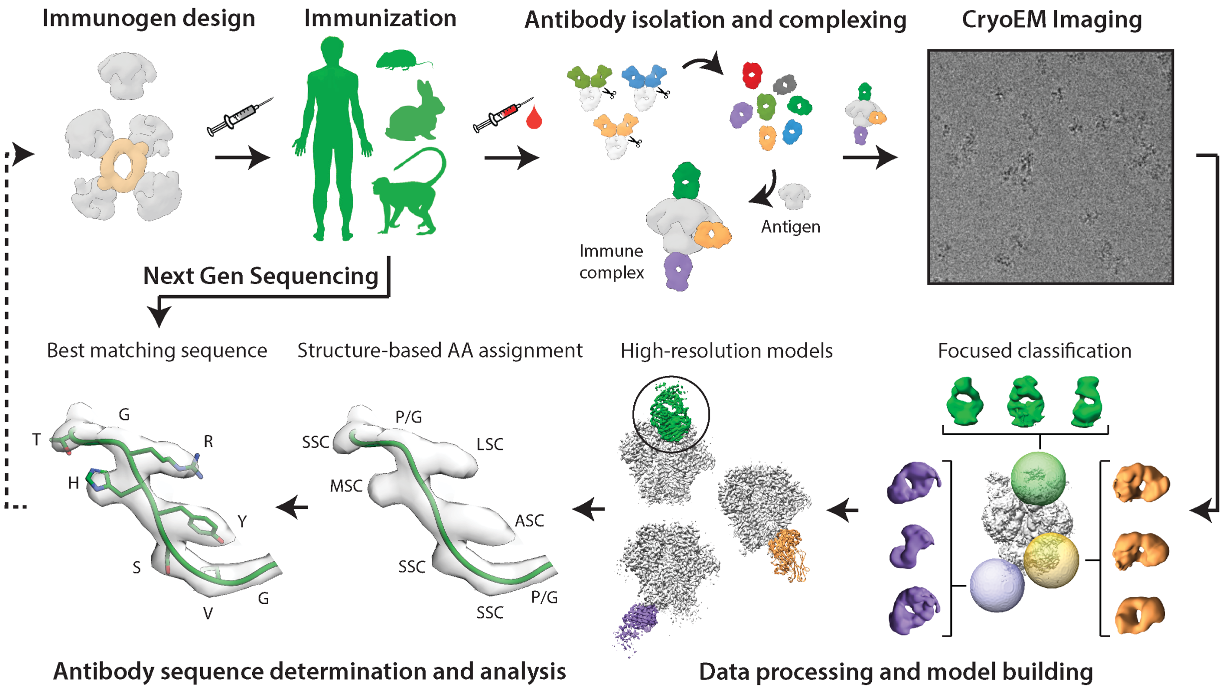

Figure S4. Illustration of the method used for antibody sequence determination.

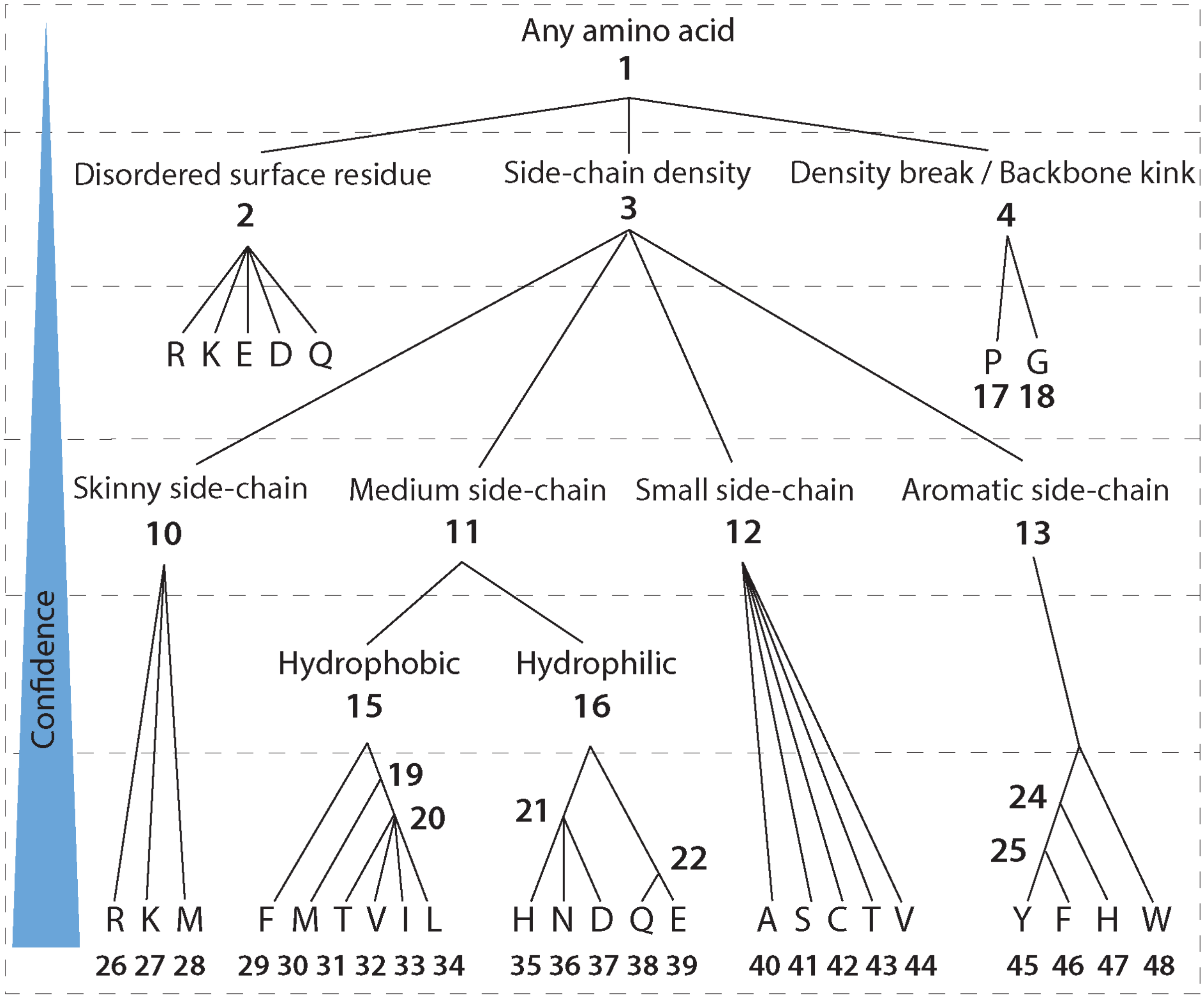

Figure S5. Amino acid assignment tree with numerical category identifiers. Amino acids that appear more than once under different codes (for example, Methionine can be represented as category 28 or 30) are treated as redundant entries in the search.

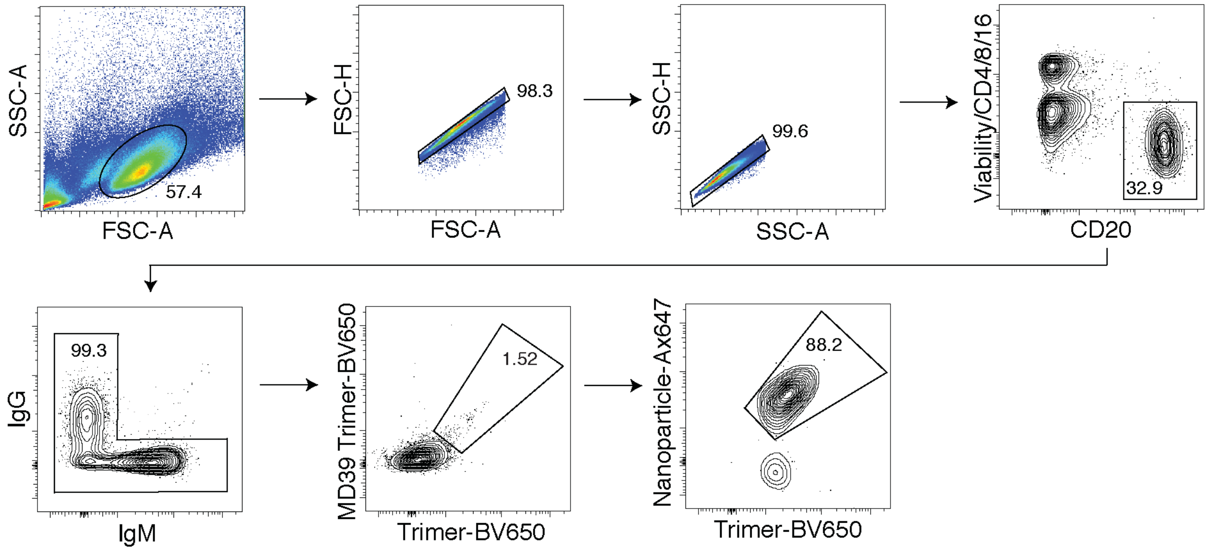

Figure S6. Overview of the gating strategy used for B-cell sorting. More detailed explanations are provided in the methods section.

A

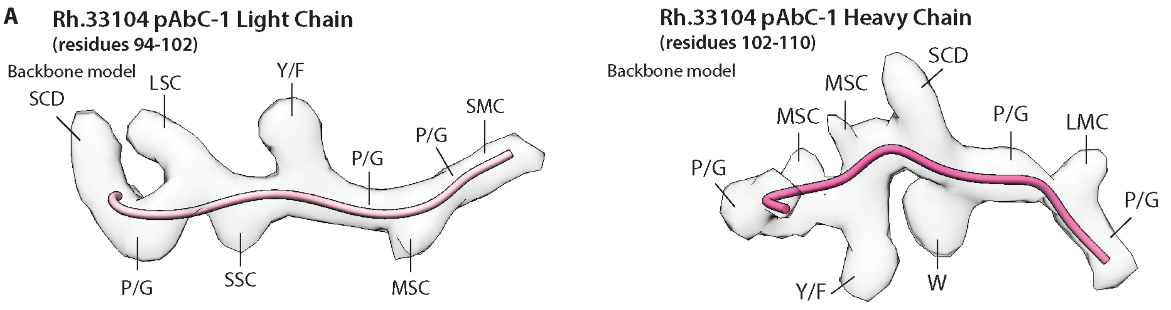

Prediction	SCD	P/G	LSC	SSC	Y/F	P/G	MSC	P/G	SMC
Matching	+	+	+	+	+	+	+	+	+
Best Match	Y	P	R	T	F	G	Q	G	T

Prediction	P/G	MSC	Y/F	MSC	SCD	W	P/G	LMC	P/G
Matching	-	+	+	+	+	+	+	+	+
Best Match	S	N	F	D	F	W	G	Q	G

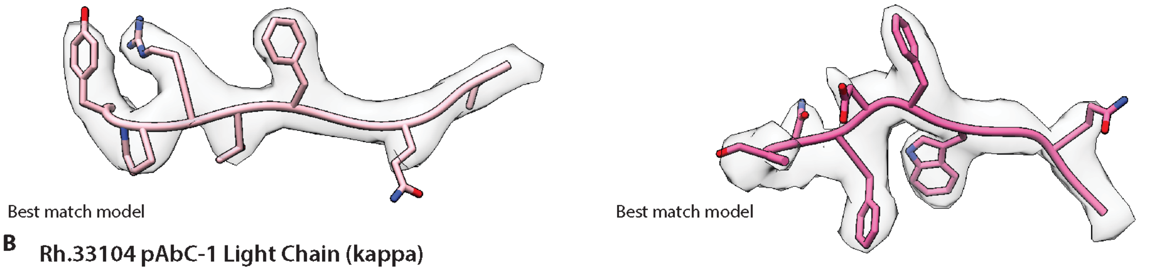

B

Rh.33104 pAbC-1 Light Chain (kappa)

Segment	Sequence and matching	Matches	Score
FR1	D I Q M T Q S P S S L S A S V G D T V T T T C R A S<br>+ +++ + + + ++ + ++ ++ + + + ++ + ++ + ++ ++	26/26	5.25
CDR1	Q D I S N D<br>+ + ++ + +	6/6	0.85
FR2	L A W Y Q Q K P G K A P K P L L Y<br>+ + + + + - + + + + + + + - + + +	15/17	5.40
CDR2	Y A S<br>+ + +	3/3	0.65
FR3	N L E S G V P S M F S G S G S G T D F T L T I S S L Q P E D F A S Y F C<br>+ + - + + + + + + + + ++ + + ++ + ++ +++++ + + + + + ++ ++ +	35/36	9.70
CDR3	Q Q Y N S Y P R T<br>+ + + ++ + ++ +	9/9	1.89
		**Total**	**94/97**	**23.74**

Rh.33104 pAbC-1 Heavy Chain

Segment	Sequence and matching	Matches	Score
FR1	Q V Q L Q E S G P G L V K P S E T L S L T C A V S<br>- + - + - - - + + - - + + + + - + ++++ + + + -	16/25	2.81
CDR1	G G S F S G Y S<br>++ + + + + + -	7/8	1.67
FR2	W G W I R Q P P G K G L E W I G S<br>+ + + ++ + + ++ + + + + + + + -	16/17	7.32
CDR2	I I G R T G S T<br>++ - + ++ + +	7/8	1.49
FR3	A Y N P S L T S R V T I S R D T S N N Q F S L K L T S L T A A D T A V Y Y C<br>+ + + + +++ + ++++++ + - + + + + + + ++ + +++ - + + - + + + + + + +	35/38	7.31
CDR3	A R Q Q S N F D F<br>+ + + + - + + + +	8/9	1.42
		**Total**	**89/105**	**22.02**

Figure S7. [A] Example of the amino-acid assignment for Rh.33104 pAbC-1. For light chain (left), residues 94-102 and the corresponding area of the map are shown. For heavy chain (right), residues 102-110 and the corresponding area of the map are shown. Models are displayed in pink and maps are represented as transparent light-gray surface. **[B]** Best sequence matches for Rh.33104 pAbC-1 light chain (top) and heavy chain (bottom). Matching to assignments at each position is shown (+/-). Overall agreement to predictions and scoring data are shown on the right.

A **Rh.33172 pAbC-2 Light Chain**
(residues 32-40)

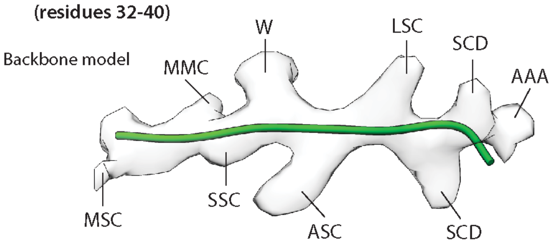

Prediction	MSC	MMC	SSC	W	ASC	LSC	SCD	SCD	AAA
Matching	+	+	+	+	+	-	+	+	+
Best Match	Q	L	C	W	Y	Q	L	K	P

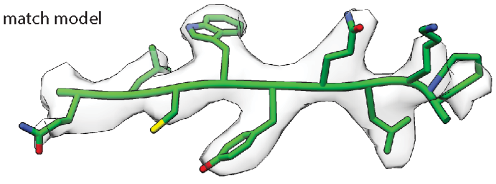

Rh.33172 pAbC-2 Heavy Chain
(residues 107-115)

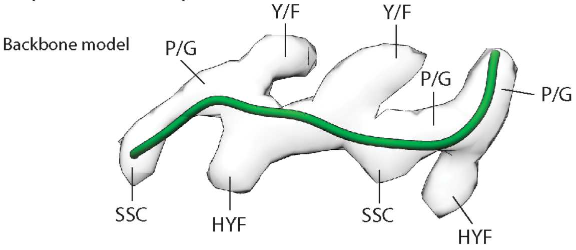

Prediction	SSC	P/G	Y/F	HYF	Y/F	SSC	P/G	HYF	P/G
Matching	-	+	+	+	+	+	+	+	+
Best Match	D	G	Y	H	Y	T	G	Y	G

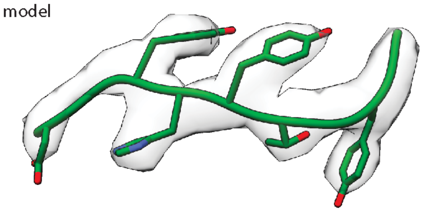

B

Rh.33172 pAbC-2 Light Chain (kappa)

Segment	Sequence and matching	Matches	Score
FR1	D I Q M T Q S P S S L S A S I G D R V T V T C R A S<br>+ - + - + - - + + + - + + - + + - + + + + - + + + +	18/26	3.12
CDR1	Q G I N M Q<br>+ + + + + +	6/6	1.03
FR2	L C W Y Q L K P G K A P T L L I Y<br>+ + + + - + + + + + + - + + + + +	15/17	3.32
CDR2	G T S<br>+ + +	3/3	0.90
FR3	G L Q T G V S S R F S G S G S G T N F T L T I S S L Q P E D V A T Y Y C<br>- + + + + + - + + + + + + + - - + + - + + + + + + + + + + + + + + - + + + +	30/36	5.22
CDR3	Q Q D Y T T P F T<br>+ + + + + + - + +	8/9	1.03
	Total	**80/97**	**14.62**

Rh.33172 pAbC-2 Heavy Chain

Segment	Sequence and matching	Matches	Score
FR1	Q V Q L V Q S G A E V K M P G T S V K L S C K T S<br>+ + + + + - + + + + + - + + + + ++ ++ + ++ ++	23/25	3.94
CDR1	G Y T F T S Y N<br>+ + + + + + + -	7/8	1.62
FR2	I N W V R Q A P G Q A L E W M G W<br>+ - + + + + + + - + + + + + + + + +	15/17	4.88
CDR2	I N P N N G T T<br>+ + + + + + + ++	8/8	1.16
FR3	D Y A Q K F Q G R V T M T R D T S T T T A Y M Q L N S L R S E D T A V Y Y C<br>+ + + - + + + + + + + + + - - + - + + + + + + + + + - + + + ++ + + ++ ++ +	33/38	7.18
CDR3	A R A R G G Y E D D D G Y H Y T G Y G L D<br>+ + + - - + + + + - - + + + + + + + + + +	17/21	5.37
	Total	**103/117**	**24.15**

Figure S8. **[A]** Example of the amino-acid assignment for Rh.33172 pAbC-2. For light chain (left), residues 32-40 and the corresponding area of the map are shown. For heavy chain (right), residues 107-115 and the corresponding area of the map are shown. Models are displayed in green and maps are represented as transparent light-gray surface. **[B]** Best sequence matches for Rh.33172 pAbC-2 light chain (top) and heavy chain (bottom). Matching to assignments at each position is shown (+/-). Overall agreement to predictions and scoring data are shown on the right.

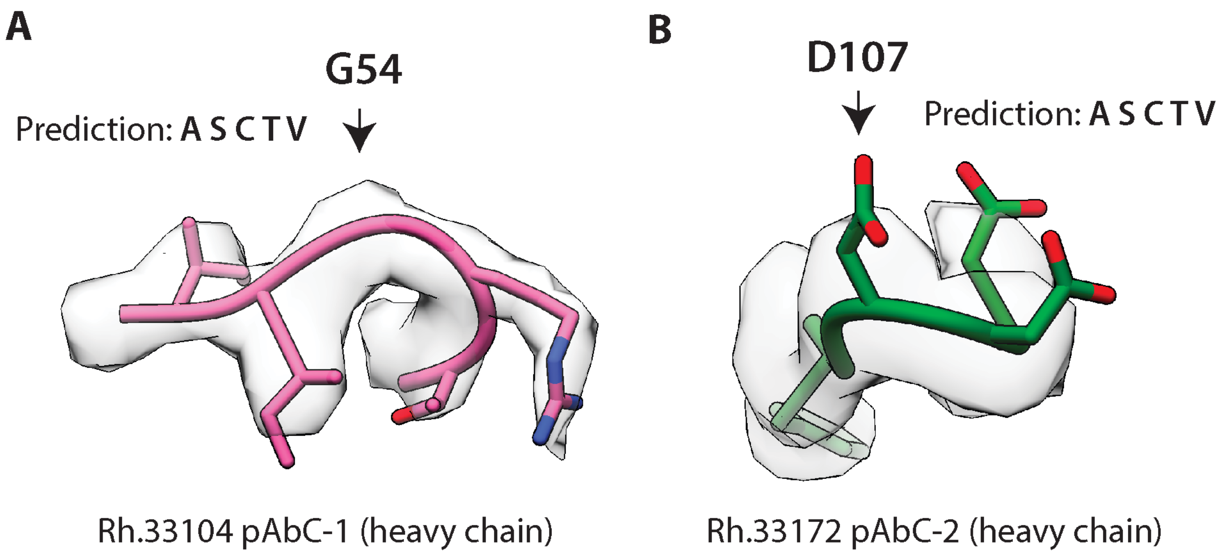

Figure S9. Examples of the most common mismatches observed in the Rh.33104 (pink) and Rh.33172 searches (green). **[A]** Small side-chain category (amino acids: A, S, C, T, V) was predicted based on structural data but P/G category (amino acids: P and G) was in the sequence. **[B]** Small side-chain category (amino acids: A, S, C, T, V) was predicted based on structural data but medium side-chain LMC category (amino acids: H, N, D, Q, E) was in the sequence. Map segments are represented as transparent light-gray surface.

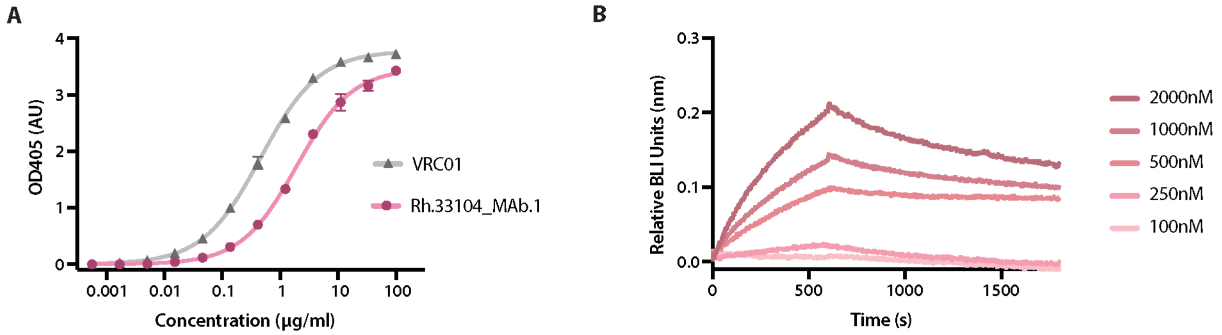

	Rh.33104 mAb.1	VRC01
EC50 (µg/ml)	1.93	0.46

	kon (1/M*s)	koff (1/s)	Kd (M)
Rh.33104 mAb.1	$5.29 * 10^2$	$4.76 * 10^{-4}$	$(8.9 \pm 0.6) * 10^{-7}$

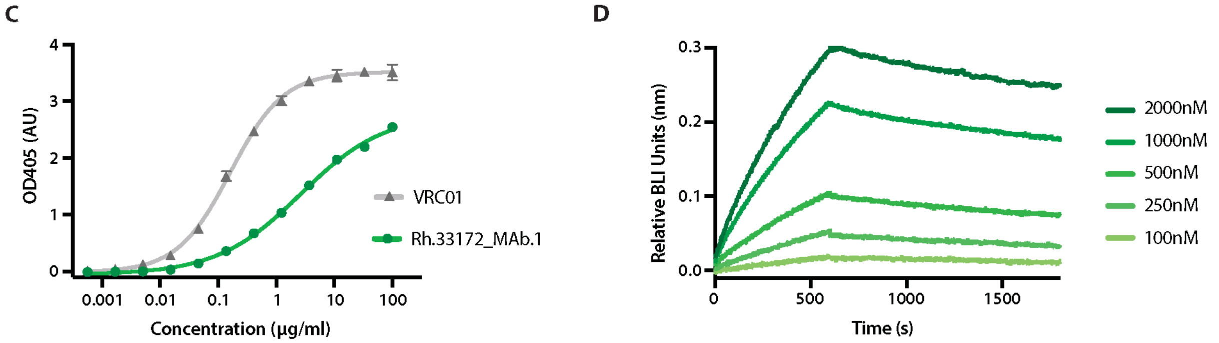

	Rh.33172 mAb.1	VRC01
EC50 (µg/ml)	2.64	0.16

	kon (1/M*s)	koff (1/s)	Kd (M)
Rh.33172 mAb.1	$5.03 * 10^2$	$9.08 * 10^{-5}$	$(1.80 \pm 0.03) * 10^{-7}$

Figure S10. Binding data for Rh.33104 mAb.1 and Rh.33172 mAb.1. Sandwich ELISA was used to quantify the interaction of BG505 SOSIP to the IgG versions of Rh.33104 mAb.1 **[A]** and Rh.33172 mAb.1 **[C]**. VRC01 IgG was used as a positive control and reference. EC_{50} values are in the tables below the corresponding graphs. BLI was used to determine the kinetic binding parameters of the interaction between BG505 SOSIP and the Fab versions of Rh.33104 mAb.1 **[B]** and Rh.33172 mAb.1 **[D]**. Antigen concentrations used to generate each binding curve are illustrated on the right in panels **[B]** and **[D]**, while the corresponding kinetic parameters are presented in the table below each graph.

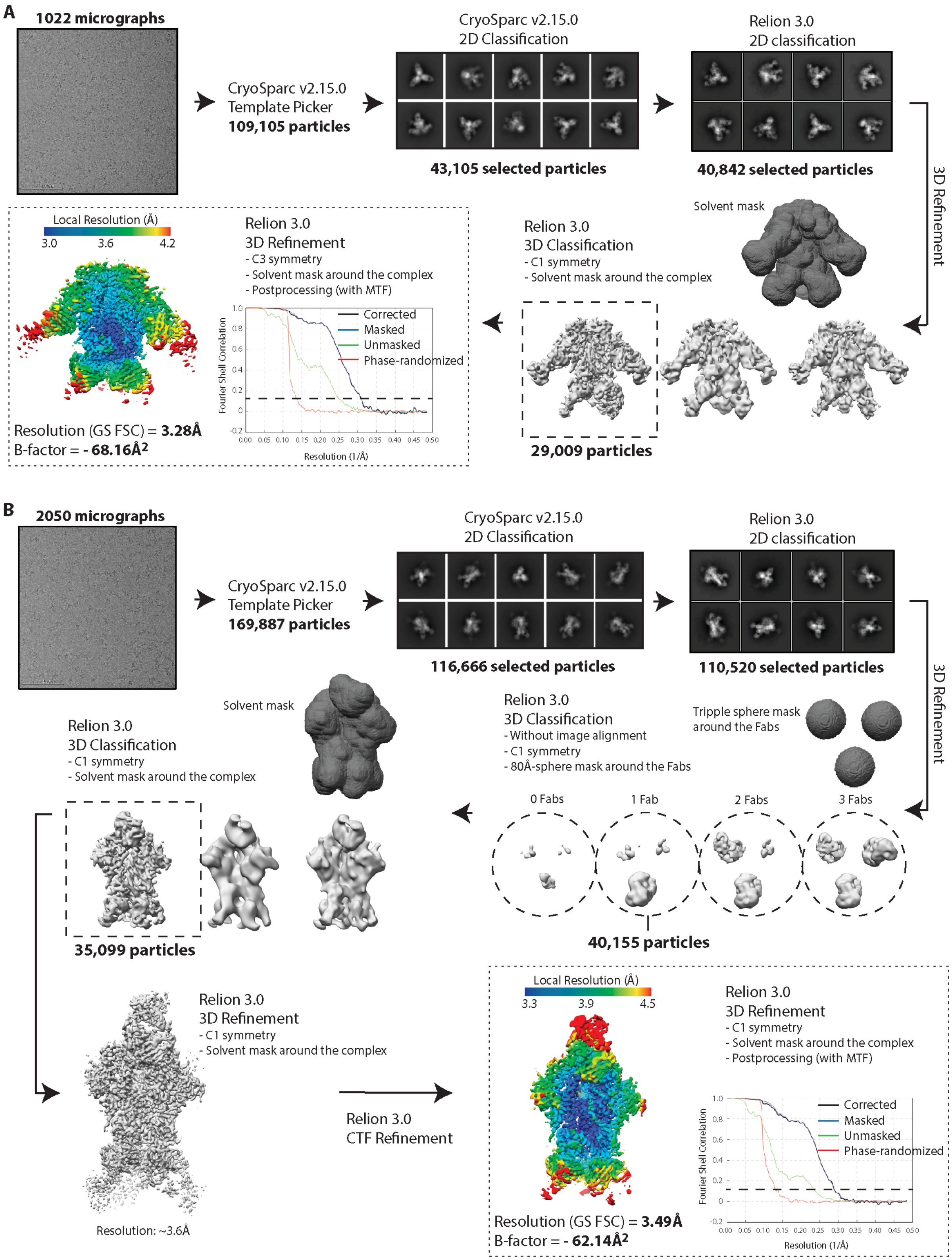

Figure S11. Schematic representation of the data processing workflow for cryoEM data with relevant statistics. The samples were **[A]** BG505 SOSIP complexed with Rh.33104 mAb.1 and RM20A3 and **[B]** BG505 SOSIP complexed with Rh.33172 mAb.1 and RM19R
